# Data transformation and model selection in bivariate allometry

**DOI:** 10.1242/bio.060587

**Published:** 2024-09-16

**Authors:** Gary C. Packard

**Affiliations:** Department of Biology, Colorado State University, Fort Collins, CO 80523, USA

**Keywords:** Allometry, Model selection, Nonlinear regression, Scaling, Fiddler crabs, Dobsonflies

## Abstract

Students of biological allometry have used the logarithmic transformation for over a century to linearize bivariate distributions that are curvilinear on the arithmetic scale. When the distribution is linear, the equation for a straight line fitted to the distribution can be back-transformed to form a two-parameter power function for describing the original observations. However, many of the data in contemporary studies of allometry fail to meet the requirement for log-linearity, thereby precluding the use of the aforementioned protocol. Even when data are linear in logarithmic form, the two-parameter power equation estimated by back-transformation may yield a misleading or erroneous perception of pattern in the original distribution. A better approach to bivariate allometry would be to forego transformation altogether and to fit multiple models to untransformed observations by nonlinear regression, thereby creating a pool of candidate models with different functional form and different assumptions regarding random error. The best model in the pool of candidate models could then be identified by a selection procedure based on maximum likelihood. Two examples are presented to illustrate the power and versatility of newer methods for studying allometric variation. It always is better to examine the original data when it is possible to do so.

## Teaser

**Summary:** Modern methods of nonlinear regression allow investigators to perform comprehensive studies of allometric variation without transforming the original data.

## INTRODUCTION

In analyzing data which does not match the assumptions of the conventional methods of analysis, we have two choices… We may bend the data to fit the assumptions by making a transformation. Or we may develop new methods of analysis with assumptions which fit the data in its ‘original’ form somewhat better. (Tukey, 1957)

John Tukey (1915–2000) was a world-class mathematician and statistician who noted over half a century ago that investigators have two options when they are confronted with data that fail to satisfy the assumptions underlying conventional methods for statistical analysis: (1) investigators can “bend” the data by the Procrustean expedient of transformation to create a new mathematical distribution that more nearly satisfies the assumptions; or (2) they can develop new methods that will satisfy the assumptions without altering the native form for the data (Tukey, 1957). His observation is as relevant to biologists today as it was then, as is revealed by the contemporary controversy concerning methods for conducting research on bivariate allometry. Whereas some investigators advocate for a standardized method that entails logarithmic transformation of the original data (Pélabon et al., 2018; Tsuboi, 2019; Glazier, 2021), other workers champion the use of the newer methods that were envisioned by Tukey (Packard, 2020a; 2023a). Choosing between these alternatives is not a trivial matter. Many of the conceptual models for evolution of form and function in plants and animals (e.g. the Metabolic Theory of Ecology, the Contextual Multimodal Theory) are based on perceptions of pattern in allometric variation, but the perceptions themselves commonly depend on whether data are studied in their transformed or untransformed state.

Students of allometry have used the logarithmic transformation for over a century to linearize bivariate distributions that are curvilinear on the arithmetic scale (Snell, 1892; Dubois, 1897; Lapicque, 1898). The critical assumption in such cases is, of course, that the transformed data actually follow a linear path in graphical display (Reeve, 1940; Kavanagh and Richards, 1942; Reeve and Huxley, 1945; Richards and Kavanagh, 1945). When the distribution is linear, the equation for a straight line fitted to the distribution can be back-transformed (i.e. exponentiated) to form a simple, two-parameter power function – the equation of simple allometry (Huxley and Teissier, 1936) – for describing the observations on the original scale (Huxley, 1924; 1932). Other assumptions of this “Huxlian” protocol, like constant variance and normal distribution for residuals, were not matters of wide concern to early investigators and became important only after the general issue of assumptions was taken up by statisticians in the mid-20th Century (Hoyle, 1973). A straight line fitted to transformations typically assumes homoscedasticity (i.e. constant variance for the response variable at all levels for the predictor) and a normal distribution for residuals whereas the two-parameter power equation formed by back-transformation necessarily assumes heteroscedasticity and lognormality on the original scale (Xiao et al., 2011).

**Fig. 1.**
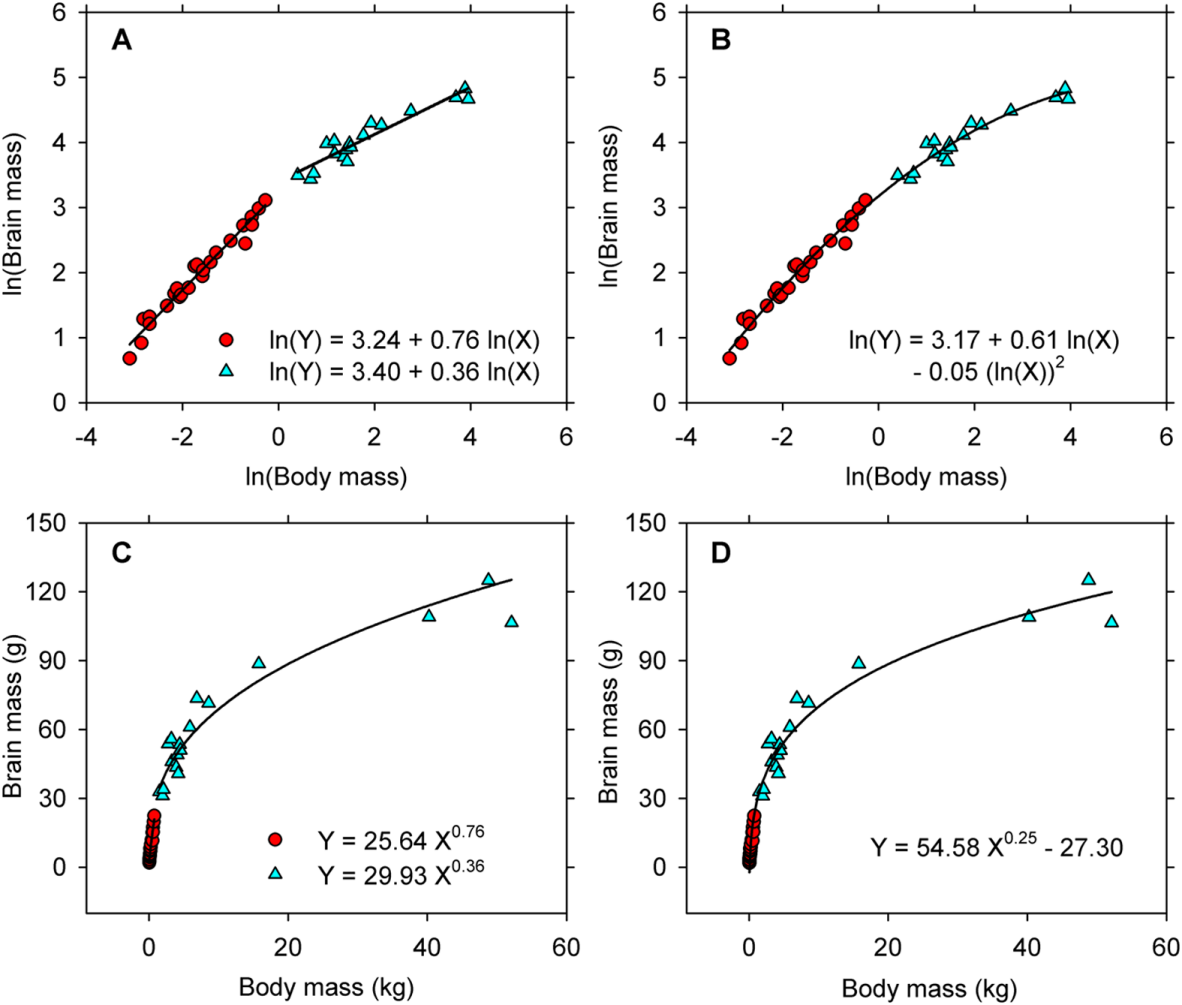
**Data for brain-body allometry in an ontogenetic series of 42 sheep (*Ovis aries*) illustrate both biphasic, loglinear allometry (A) and complex allometry (B).** (A) Putative regions of rapid relative growth (red circles) and slow relative growth (cyan triangles) were identified by piecewise regression (see Packard, 2021a). (B) The full sample is described equally well in logarithmic domain by a quadratic equation that characterizes complex allometry. (C) The lines fitted to quasi-linear parts of the bivariate distribution in panel A indicate that a pair of two-parameter power equations is needed to describe the full distribution for the original, untransformed observations. (D) A three-parameter power equation fitted to untransformed observations by nonlinear regression captures the pattern in the full distribution. No abrupt change in the pattern of relative growth occurs at some inflexion point (contra panel A), nor is a pair of simple power equations needed to capture pattern in the full distribution (contra panel C). Also, the exponent does not change continuously as a function of body size (contra expectations based on panel B). (Figure modified from Packard, 2021a.)

The ease in implementation and apparent simplicity of the Huxlian protocol have led to its adoption as the standard method for conducting allometric research (Smith, 1980, 1984). Data typically are transformed to logarithms without being inspected first in their native state, and the transformations then are fitted with a straight line. The fitted equation usually is interpreted in the context of untransformed data [i.e. variation in Y relative to X, not in ln(Y) relative to ln(X)]. If curvature is detected in the bivariate plot, the distribution often is fitted with two (or sometimes three) straight lines (“biphasic, loglinear allometry”; Fig. 1A) or with a quadratic polynomial (“complex allometry”; Fig. 1B). The two straight lines of biphasic allometry are taken to mean that the pattern of variation changes abruptly at some inflexion point in the distribution for the predictor variable (Strauss, 1993). A quadratic polynomial, on the other hand, is interpreted to mean that the exponent in a simple, two-parameter power equation for describing the untransformed data is itself a function of the predictor (Strauss, 1993).

However, biphasic and polyphasic allometry are statistical artifacts that result from attempts to make Huxley's approach work in situations for which it is ill suited (Bales, 1996; Sartori and Ball, 2009; Packard, 2015; 2016; 2017a; 2017b; 2019; 2021a; 2023b). For example, the straight lines fitted to quasi-linear parts of the distribution in biphasic allometry indicate that two different, two-parameter power equations (both of which pass through the origin) are needed to describe pattern in the full distribution for untransformed observations (Fig. 1C). Complex allometry, on the other hand, translates into an uninterpretable “two-parameter” power equation with three parameters and two predictor variables [i.e. Y=*β_0_*×X^(*β1*+(*β2*×ln(X)))^] for describing observations on the original scale (Sartori and Ball, 2009; MacKay, 2011; Packard, 2017b; 2023b). In both biphasic and complex allometry, the distribution for the untransformed observations is described explicitly in terms of two-parameter power equations, yet data in such cases typically are characterized quite well by a single, three-parameter power function that spans the full range in the predictor variable (Fig. 1D). This means, of course, that the best equation does not pass through the origin of a bivariate graph and that the curvilinearity in logarithmic domain is caused by the non-zero intercept (Sartori and Ball, 2009; Packard, 2016; 2017b; 2019; 2021a; 2023b). These points reaffirm that the only legitimate application of the Huxlian protocol is to bivariate distributions that are linear in logarithmic domain and that are described by a two-parameter power function on the arithmetic scale (Reeve, 1940; Kavanagh and Richards, 1942; Reeve and Huxley, 1945; Richards and Kavanagh, 1945). Bivariate distributions that deviate from log-linearity need to be studied on the untransformed scale by the newer methods anticipated by Tukey (Packard, 2020a; 2023a).

**Fig. 2.**
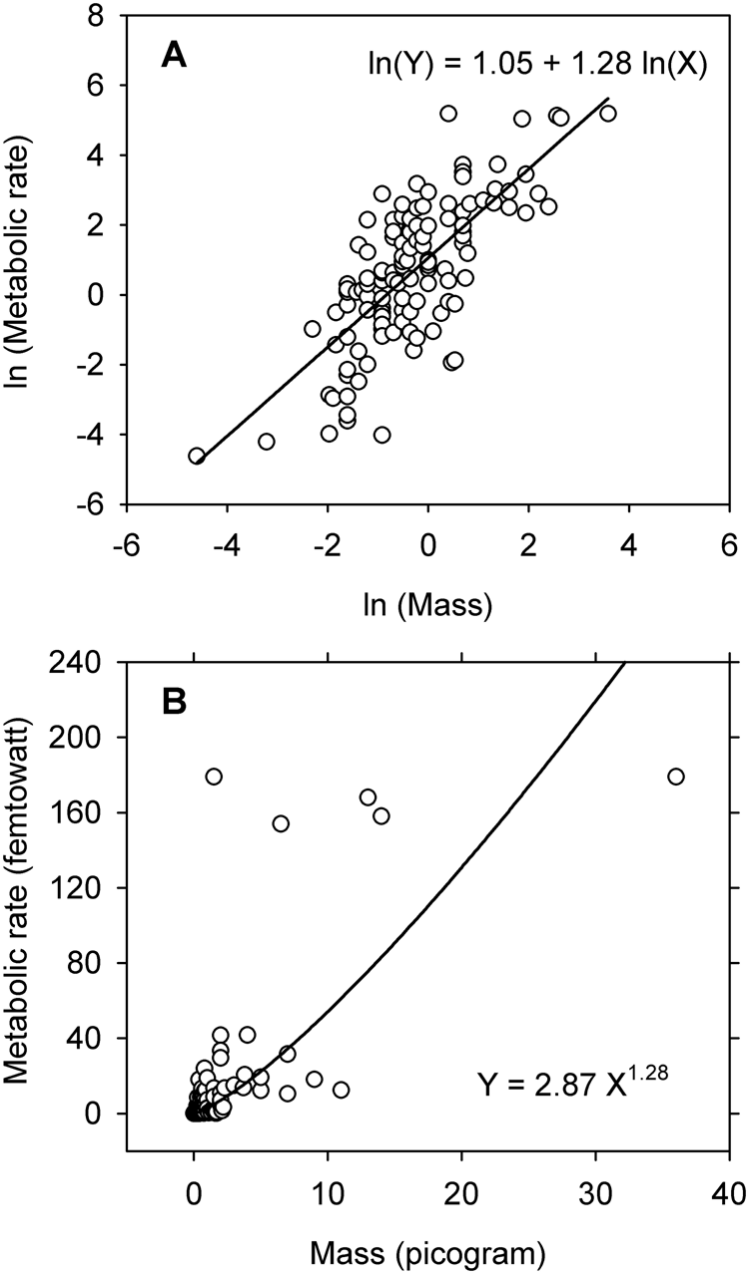
**DeLong et al. (2010)** **evaluated the metabolic allometry of unfed prokaryotes as part of an investigation into evolutionary transitions among levels of biological organization**. The data described here have been rescaled from the original to facilitate reporting. (A) The allometric relationship between ln(metabolic rate) and ln(mass) follows the path of a straight line fitted by ordinary least squares. (B) Most observations for untransformed data are clustered in the lower left of the graphical display but five observations lie in a horizontal band located well above all the other observations. The model estimated by back-transformation fails to identify a pattern in the observations because no pattern is apparent. (Figure modified from Packard, 2011.)

The Huxlian protocol works well in many situations where bivariate data are loglinear, but it does not work well in others. Unfortunately, occasions on which the protocol works poorly often go undetected because model validation is typically done only on logarithms. Data and models that look reasonably good on the logarithmic scale (Fig. 2A) can look rather ugly when re-expressed in arithmetic form (Fig. 2B). Even when the re-expressed model looks good in the arithmetic domain, it may not be the only model (or even the best model) for describing the data in question (Packard, 2020b,c). This insidious problem has the potential to introduce ambiguity into analyses and thereby impact emerging “theories” for the evolution of form and function in both plants and animals.

## RESULTS

### Growth by the crusher claw in fiddler crabs

Consider the data gathered by Dinh (2022) on ontogenetic development of the crusher claw in males of the fiddler crab *Uca pugilator*. His assessment of growth by the claw was part of a comprehensive investigation of the “cost-minimization hypothesis” for growth and maintenance of a sexually selected structure that is used in display or combat.

**Fig. 3.**
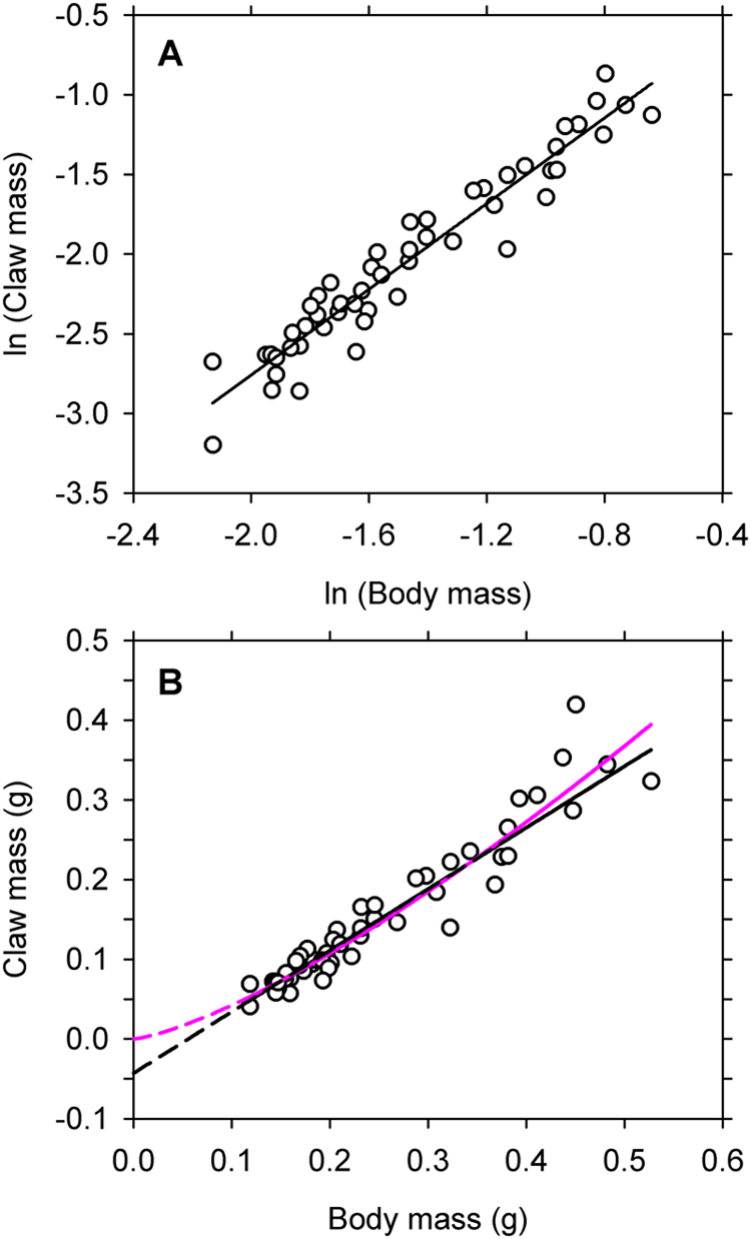
**Dinh (2022)** **examined allometric variation in size of the enlarged crusher claw in 51 males of the fiddler crab *Uca pugilator*.** (A) Natural logarithms for claw mass versus body mass are well described by a straight line (Table 1). (B) A straight line with non-zero intercept (black) and a two-parameter power equation (pink) capture near-identical amounts of information from the data (Table 2). The dashed portions of each line are extrapolations beyond the range of the data.

I downloaded measurements for dry mass of the body exclusive of the crusher claw, and for dry mass of the excised claw itself, from the online supplement to the original paper (Dinh, 2022). Logarithmic transformations of data for the 51 males appear to follow the path of a straight line in bivariate display, so I fitted a linear polynomial to the distribution (Fig. 3A). However, I also fitted a quadratic equation to the observations to assess the possibility of a cryptic departure from linearity. Both the models satisfied assumptions for homoscedasticity and normality (Table 1). Log-likelihood values indicate that the two models capture virtually identical amounts of information in the data, so the quadratic (with its additional parameter) can be dismissed on grounds of overfitting (Burnham and Anderson, 2002). The straight line explains 92% of the variation in the response variable (Fig. 3A). The slope for the line is 1.34 (95% confidence interval=1.24–1.45 by likelihood ratio). When the equation is re-expressed on the arithmetic scale it becomes a two-parameter power equation with an exponent of 1.34 (Fig. 3B).

**Table 1.**
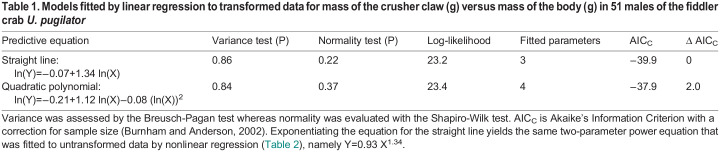
Models fitted by linear regression to transformed data for mass of the crusher claw (g) versus mass of the body (g) in 51 males of the fiddler crab *U. pugilator*

**Table 2.**
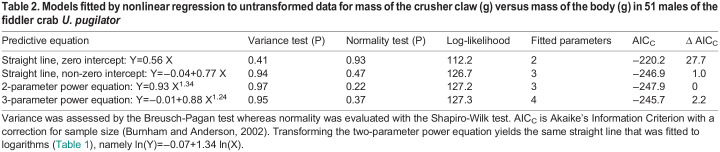
Models fitted by nonlinear regression to untransformed data for mass of the crusher claw (g) versus mass of the body (g) in 51 males of the fiddler crab *U. pugilator*

The preceding finding is based on a single model fitted to logarithmic transformations, because no alternative model can be legitimately fitted to the observations using the conventional approach. However, nonlinear regression on untransformed observations provides a way to fit a two-parameter power equation as well as alternative functions for describing the original bivariate distribution (Packard, 2020a; 2023a). In the present case, a bivariate graph of untransformed data somewhat surprisingly invites a comparison of the two-parameter power equation with an equation for a straight line (Fig. 3B). I accordingly fitted two straight lines and a two-parameter power function to the original data using the Model Procedure for SAS 9.4 (SAS Institute, 2004). One of the straight lines passed through the origin, and the other had an explicit, non-zero intercept. I also fitted a three-parameter power equation to determine whether the simple power equation could be improved by adding a non-zero intercept.

Full information maximum likelihood (FIML) was used to estimate the best values for parameters in the specified models. The error structure was modeled in all cases as lognormal and heteroscedastic (Packard, 2020a; 2023a), with residuals computed as ln (observed Y/ predicted Y) (https://support.sas.com/documentation/cdl/en/etsug/60372/HTML/default/viewer.htm#etsug_model_sect045.htm). The protocol consequently replicated the assumptions of a simple power equation estimated by back-transforming the equation for a straight line fitted to logarithms (Packard, 2020a; 2023a). All four models fitted to the arithmetic distribution satisfied assumptions for variance and normality (Table 2). Quality of the fits was then assessed using Akaike's Information Criterion with a correction for sample size (Burnham and Anderson, 2002). Models with AIC_C_ less than 7–8 were judged to be worthy of consideration (Richards, 2005; Burnham et al., 2011; Richards et al., 2011).

Note, first, that the two-parameter power equation fitted to the original data by nonlinear regression is identical to the two-parameter power equation formed by back-transforming the straight line fitted to logarithms (Tables 1,2). Conversely, the equation for a straight line formed by transforming the power equation is identical to the equation for the straight line fitted to logarithms (Tables 1,2). Thus, despite claims to the contrary (Pélabon et al., 2018; Tsuboi, 2019; Glazier, 2021), logarithmic transformation is not necessary in an analysis of allometric variation, because the same functional equation, with the same assumptions about form for error, can be fitted directly to the original data without transforming the observations. Indeed, the only difference between models estimated by the Huxlian approach and by nonlinear regression is that the sign for residuals in the model fitted by nonlinear regression is reversed by the computational algorithm. In both instances, the model predicts geometric means (not arithmetic means) for mass of the claw (SAS Institute, 2004, p. 1110 ff), so a “correction” needs to be applied if the model is to be used to describe arithmetic means for the response variable (Hayes and Shonkwiler, 2006). The correction typically is minor, and perceptions of pattern are unaffected.

Second, the straight line through the origin is not well supported by AIC_C_, but the other three models capture virtually identical amounts of information in the data (Table 2). However, one additional parameter was estimated for the three-parameter power function, so it can be dismissed on grounds of overfitting (Burnham and Anderson, 2002). The other two models are equivalent on the basis of the numerical treatment. Of course, the exponent for the straight line is 1 whereas that for the simple power equation is 1.34 (95% confidence interval=1.24–1.45 by likelihood ratio).

A graph of the competing models is informative (Fig. 3B). First, the path of the two-parameter power equation depends importantly on the requirement that the line pass through the origin and on the presence of a high-leverage observation for a large crab (Fig. 3B). By eye, the data follow a largely linear path over the range in the distribution, so the existence of curvature might be questioned despite the equivalence of the power model with the one for a straight line (Anscombe, 1973).

The straight line, on the other hand, has a negative Y-intercept (Table 2), which means that the extrapolated line intersects the X axis at 0.05 (Fig. 3B). Such a value for the X-intercept indicates that the claw probably does not have a discernable mass before the body has attained a mass of about 50 mg. Given the small size of “first crabs” at the end of larval life, this is not an unreasonable prospect.

The pattern of variation between claw and body is positively allometric regardless of which of the competing models is considered. In the case of the power equation, the cause is the exponent exceeding 1; in the case of the straight line, the cause is the negative value for the Y-intercept (Gould, 1966). Within the range for the data, however, the straight line implies a pattern of isometric (proportional) growth (Huxley, 1932, p. 241; Thompson, 1945) whereas the power equation invokes allometric (disproportional) growth (Huxley, 1932). A decision about which model is to be accepted (if a decision is to be made at all) will necessarily be based on subjective interpretation of the graphical display of the original measurements (Anscombe, 1973). In either case, the fitted equation will provide an empirical description having no foundation in theory (Nijhout and McKenna, 2017).

### The postocular flange in dobsonflies

Ramírez-Ponce et al. (2017) studied allometric variation in size of the postocular flange in dobsonflies of the genus *Platyneuromus* (Insecta, Megaloptera). Here I concentrate on scaling of mesial width (MW) of the flange in relation to interantennal distance (IAD) in 57 males of *P. honduranus*. Mesial width is a measure of size of the flange whereas IAD is a measure of size of the head (and body?) that bears the flange. The paired flanges may have a role in visual displays by males while courting females and/or in aggressive encounters with conspecific males. Dobsonflies undergo a complete metamorphosis, so size of the postocular flange does not change after the transition from pupa to adult form. Allometric variation consequently is static. My new treatment of the data introduces a complex but interesting twist to the story while continuing to emphasize the importance of selecting among multiple models fitted to observations expressed in their native state.

**Fig. 4.**
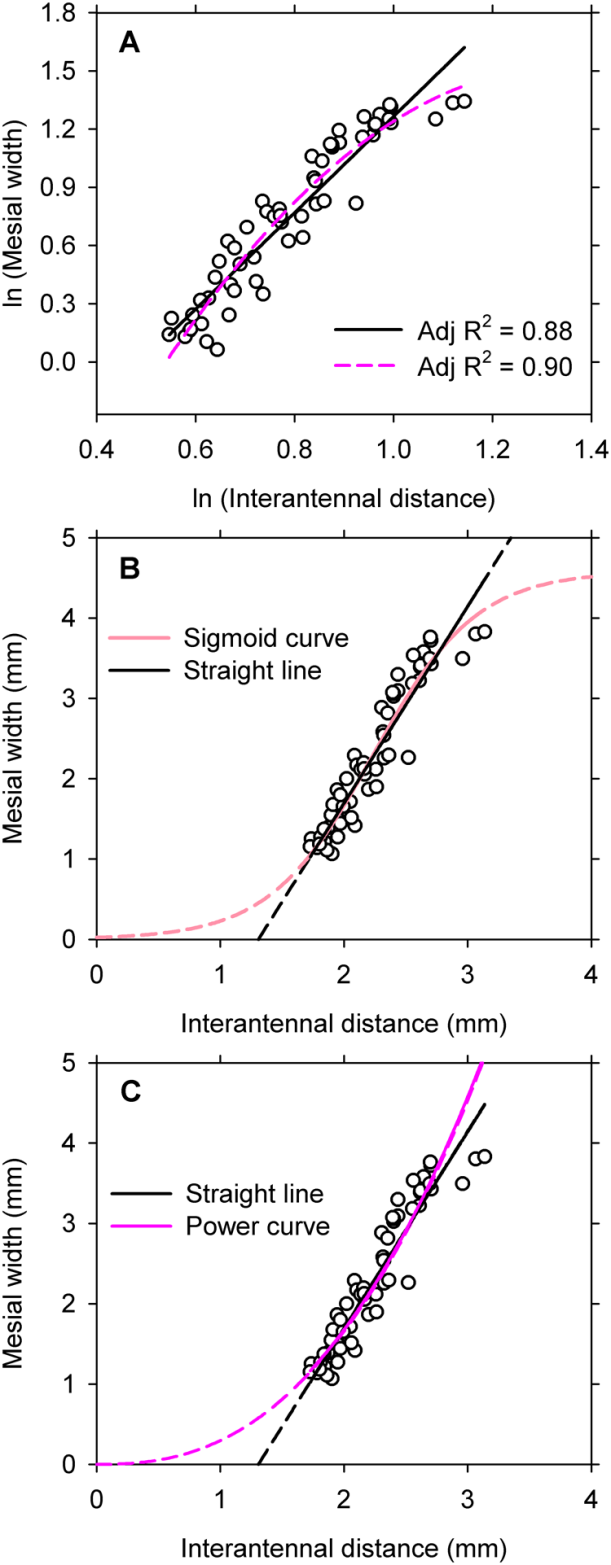
**Ramírez-Ponce et al. (2017)** **assessed allometric variation in size of the postocular flange on 57 males of the dobsonfly *Platyneuromus honduranus***. (A) Natural logarithms of mesial width of the flange versus interocular distance. The solid black line is the linear polynomial whereas the dashed pink line is the quadratic polynomial. (B) A straight line and a three-parameter sigmoid function have been fitted to untransformed observations (Table 4). Dashed lines are extrapolations beyond the range in the data. The sigmoid curve is a slightly better fit (ΔAIC=3.5), but both functions are essentially linear across the range in the data. (C) A straight line and a two-parameter power equation have been fitted to untransformed observations (Table 4). Dashed lines are extrapolations beyond the range in the data. The straight line is the better fit (ΔAIC=7.2), but both functions are essentially linear across the range in the data.

I recovered values for common logarithms from table S1 in Ramírez-Ponce et al. (2017) and then re-expressed them as natural logarithms. The plot of the logarithms fosters an impression of curvilinearity in the bivariate distribution, so I fitted a quadratic polynomial to the observations as well as a straight line (Fig. 4A). The straight line explains 88% of the variation in the response variable and the quadratic explains only slightly more (R^2^=0.90). AIC_C_ indicates, however, that the quadratic equation provides a somewhat better description for the data than does the rectilinear fit (Table 3). Residuals satisfy the test for variance, but they may deviate somewhat from normality (Table 3). Failure of the normality assumption in regression affects probability statements like confidence intervals around parameters but not estimates for the parameters themselves (Finney, 1989). Thus, the deviation from normality is not a major issue. The apparent curvilinearity in the distribution implies that the best function for describing the original, untransformed distribution is a three-parameter power equation with lognormal, heteroscedastic error (Sartori and Ball, 2009; Packard, 2017b; 2023b).

**Table 3.**
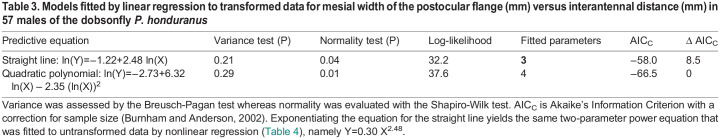
Models fitted by linear regression to transformed data for mesial width of the postocular flange (mm) versus interantennal distance (mm) in 57 males of the dobsonfly *P. honduranus*

**Table 4.**
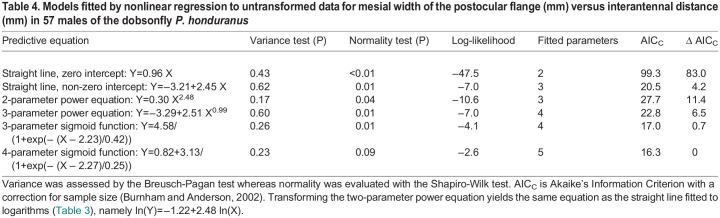
Models fitted by nonlinear regression to untransformed data for mesial width of the postocular flange (mm) versus interantennal distance (mm) in 57 males of the dobsonfly *P. honduranus*

Next, I displayed the untransformed observations on a graph with linear scaling (Fig. 4B). The observations follow a generally linear path, but a slight concave curvature may be present in the upper part of the distribution (Fig. 4B). I therefore fitted six different models to the data. First, I fitted two models for straight lines, with one passing through the origin and the other having an explicit Y-intercept. Two- and three-parameter power equations were fitted next, because a two-parameter function is estimated by the Huxlian method and a three-parameter equation is indicated by the quadratic polynomial fitted to logarithms (Sartori and Ball, 2009; Packard, 2017b; 2023b). Finally, three- and four-parameter sigmoidal functions were fitted in an attempt to capture the slight, S-shaped curvature that appears in the upper part of the distribution (Fig. 4B). All the models assumed lognormal, heteroscedastic error. Residuals for the six models satisfied the test for variance but they also deviated slightly from normality (Table 4). The departures from normality are not of major concern, for reasons already given. The models then were compared by AIC_C_ and graphical analysis.

Five important points emerge from the analyses:

(1) The four-parameter sigmoid model is the best fit by AIC_C_, but the three-parameter sigmoid model captures almost as much of the information in the data (Table 4). Thus, including a Y-intercept in the equation does not yield an appreciable improvement over the fit of the simpler, three-parameter model (ΔAIC_C_=0.7; Table 4). The four-parameter model consequently is overfitted (Burnham and Anderson, 2002) and can be removed from further consideration. The three-parameter sigmoid model is accepted here as the best model in the pool of candidate models.
(2) Both log-likelihood and AIC_C_ indicate that the straight line passing through the origin is a poor model for the data (Table 4). Any one of the other models in the pool of candidate models would be a better choice. This straight line also can be removed from further consideration.
(3) The model for the three-parameter power equation captures as much of the information in the data as does the straight line with an explicit intercept (Table 4). However, the power equation requires an additional parameter (Table 4), and coefficients for the power equation are essentially the same as those for the straight line (where the exponent is implicitly 1). Thus, the three-parameter power equation can be removed from further consideration on grounds that it is no more than a poor approximation to a straight line with an explicit Y-intercept.
(4) The model for the two-parameter power equation is identical to the model that is estimated by the Huxlian protocol and for that reason is considered here despite its high AIC_C_ relative to both the three-parameter sigmoid model (ΔAIC_C_=10.7) and the straight line with intercept (ΔAIC_C_=7.2). Note that the power equation is essentially linear over the range in the data (Fig. 4C) and that the deviation from linearity is caused by the requirement for the function to pass through the origin (Fig. 4C). This interpretation is supported by the fit of the three-parameter power equation, which yields an improvement on the two-parameter function simply be adding the Y-intercept (ΔAIC_C_=4.9).
(5) The three-parameter sigmoid model does not capture substantially more of the information in the data than does the model for a straight line with an explicit Y-intercept (ΔAIC_C_=3.5). Moreover, the sigmoid curve is essentially linear over the range in the data and does not manifest curvilinearity until it approaches the upper limit for the distribution (Fig. 4B). The sigmoid model requires one more parameter than does the straight line, so the weight of evidence slightly favors the straight line as the better fit. A new analysis of a larger data set will be needed, however, to resolve this matter fully.

Thus, two models are acceptable fits to the data: the sigmoid function passing through the origin and the straight line with a non-zero intercept. The functional equations have different implications, however. For example, the straight line posits proportional change over the range in the data (Thompson, 1945) whereas the sigmoid equation suggests a more complex pattern of variation (see Packard, 2021b). A larger pool of potential models for untransformed data, when coupled with a good protocol for selecting among competing models and simple graphical displays, leads to a better outcome than was achieved in the original investigation.

## DISCUSSION

D'Arcy Wentworth Thompson (1860–1948), who was arguably the foremost mathematical biologist of his day, was not impressed by Huxley's procedure for allometric analysis, because many of the data sets examined by Huxley could have been described just as well by straight lines fitted to the original, untransformed observations (Thompson, 1945). Unfortunately, once Huxley had convinced himself of the general importance of an analytical protocol based on logarithmic transformations (Huxley, 1950), he generally focused on transformations and didn't bother to examine observations expressed on the original scale. Had such preliminary analyses been performed, he would have discovered that transformation was not needed in many instances to “correct” a problem with the original bivariate distribution (Finney, 1989). Thompson concluded that compound interest may apply on some occasions and simple interest on others; but for this very reason, Huxley's protocol lacks general application.

In the decades following the appearance of Thompson's critique, it has become even more apparent that Huxley's approach lacks general application to problems of allometry. Many bivariate distributions fail to satisfy the fundamental requirement for linearity on the logarithmic scale (Fig. 1) and consequently cannot be legitimately examined by the Huxlian protocol. In other instances, the nonlinear transformation to logarithms obscures the actual pattern of variation in the data and causes the statistical modeling to be inaccurate and misleading (Fig. 2). Even when a two-parameter power equation estimated by the Huxlian protocol appears to be a good fit, it may not provide the only acceptable description for the observations (Figs 3,4). In any event, a good, two-parameter power model estimated by the Huxlian approach can be obtained more directly via nonlinear regression (Tables 1 versus 2, 3 versus 4).

Huxley (1932) promoted “his” protocol at a time when it was the only way by which to fit a nonlinear model (i.e. a model that is nonlinear in its parameters) to observations that follow a curvilinear path in the arithmetic domain. However, the situation has changed substantially since he wrote in 1932, and nonlinear equations with different forms for random error can now be fitted directly to untransformed data (SAS Institute, 2004, p. 999 ff). Failure to identify the best (or even a good) model for describing pattern in a bivariate distribution may have profound consequences, because many concepts for ontogenetic, static, and evolutionary allometries are based on perceptions for pattern in the data. When those perceptions are based on analyses by the Huxlian method, they have an unacceptably high probability of being weakly supported or wrong.

## Supplementary Material

10.1242/biolopen.060587_sup1 Supplementary information
