# Supplementary material for: Data transformation and model selection in bivariate allometry
Source: Biol Open. 2024 Sep 16;13(9):bio060587. doi: 10.1242/bio.060587 (PMC11427898; doi:10.1242/bio.060587)
Supplement: Supplementary information [file biolopen-13-060587-s1.pdf]

```
data NAME;
input X      Y; /* X=IAD, Y=MW  */
log_X = log(X);
log_Y = log(Y);

datalines;

1.9560      1.4896
1.8960      1.5462
1.8000      1.1733
2.6100      3.2210
2.0220      2.0008
2.0890      1.4176
3.0650      3.8004
2.3260      2.2555
2.3630      2.2933
1.8450      1.2156
1.9120      1.6771
2.4380      3.0946
2.0860      2.2896
1.9500      1.2734
2.3150      2.5816
2.4020      3.0227
2.4360      3.2979
2.6450      3.5803
2.6180      3.3891
2.1680      2.0551
2.1050      2.1696
2.0510      1.7150
1.7840      1.1387
1.7360      1.2519
1.9040      1.0651
1.8710      1.3901
2.5620      3.5358
2.3060      2.8844
2.6210      3.4092
1.8640      1.1096
2.7040      3.7215
2.3220      2.5399
1.9940      1.6548
2.4010      3.0609
2.2580      2.1173
3.1380      3.8315
2.7070      3.4280
2.5540      3.1830
1.9450      1.8617
2.6990      3.7630
1.8120      1.2729
1.8410      1.3733
2.9600      3.4947
1.7280      1.1509
2.6940      3.4955
2.1990      1.8650
2.3540      2.8173
1.8040      1.1842
1.9720      1.7988
2.1360      2.1160
```

Biology Open • Supplementary information

```
2.1590      2.1975
2.1630      2.1245
1.9710      1.4433
2.3940      3.0739
2.5210      2.2630
2.0600      1.5124
2.2640      1.8984
;

/*ods graphics off;*/


/*  STRAIGHT LINE FITTED TO LOGARITHMIC TRANSFORMATIONS  */
proc model data=NAME PRL=both;
parms a=1 b=1;
log_Y = a + b*(log_X);
fit log_Y/ FIML normal white breusch=(1 log_X);
run;

/*  QUADRATIC POLYNOMIAL FITTED TO LOGARITHMIC TRANSFORMATIONS  */
proc model data=NAME PRL=both;
parms a=1 b=1 c=1;
log_Y = a + b*(log_X) + c*(log_X)**2;
fit log_Y/ FIML normal white breusch=(1 log_X);
run;



/*  STRAIGHT LINE (NO INTERCEPT) FITTED TO RAW DATA; LOGNORMAL,
HETEROSCEDASTIC ERROR  */
proc model data=NAME method=Marquardt PRL=both iter=100 converge=0.001;
Y = b*X;
resid.Y = log (actual.Y/pred.Y);
fit Y/ FIML normal white breusch=(1 X) out = output1 outpredict outresid;
run;
/***proc print data=output1;
run;
***/

/*  STRAIGHT LINE (WITH INTERCEPT) FITTED TO RAW DATA; LOGNORMAL,
HETEROSCEDASTIC ERROR  */
proc model data=NAME method=Marquardt PRL=both iter=100 converge=0.001;
Y = a + b*X;
resid.Y = log (actual.Y/pred.Y);
fit Y/ FIML normal white breusch=(1 X) out = output2 outpredict outresid;
run;
/***proc print data=output2;
run;
***/

/*  2-PARAM POWER FUNCTION FITTED TO RAW DATA; LOGNORMAL, HETEROSCEDASTIC
ERROR  */
proc model data=NAME method=Marquardt PRL=both iter=100 converge=0.001;
parms a=1 b=1;
Y = a * X**b;
resid.Y = log (actual.Y/pred.Y);
fit Y/ FIML normal white breusch=(1 X) out = output3 outpredict outresid;
```

Biology Open • Supplementary information

```
run;
/***proc print data=output3;
run;
***/

/*  3-PARAM POWER FUNCTION FITTED TO RAW DATA; LOGNORMAL, HETEROSCEDASTIC
ERROR  */
proc model data=NAME method=Marquardt PRL=both iter=200 converge=0.001;
parms a=0.4 b=0.7 y0=1.3;
Y = y0 + a * X**b;
resid.Y = log (actual.Y/pred.Y);
fit Y/ FIML normal white breusch=(1 X) out = output4 outpredict outresid;
run;
/***proc print data=output4;
run;
***/

/*  3-PARAM SIGMOID CURVE FITTED TO RAW DATA; LOGNORMAL HETEROSCEDASTIC ERROR
*/
proc model data=NAME method=Marquardt PRL=both iter=100 converge=0.001;
parms x0=2.15 a=4.2 b=0.36;
Y = a/(1 + exp(-(X - x0)/b));
resid.Y = log (actual.Y/pred.Y);
fit Y/ FIML normal white breusch=(1 X) out = output5 outpredict outresid;
run;
/***proc print data=output5;
run;
***/

/*  4-PARAM SIGMOIDAL CURVE FITTED TO RAW DATA; LOGNORMAL HETEROSCEDASTIC
ERROR  */
proc model data=NAME method=Marquardt PRL=both iter=100 converge=0.001;
parms y0=0.9 x0=2.3 a=3 b=0.22;
Y = y0 + a/(1 + exp(-(X - x0)/b));
resid.Y = log (actual.Y/pred.Y);
fit Y/ FIML normal white breusch=(1 X) out = output6 outpredict outresid;
run;
/***proc print data=output6;
run;
***/
```

Biology Open • Supplementary information
